# A single‐population GWAS identified *AtMATE* expression level polymorphism caused by promoter variants is associated with variation in aluminum tolerance in a local *Arabidopsis* population

**DOI:** 10.1002/pld3.250

**Published:** 2020-08-12

**Authors:** Yuki Nakano, Kazutaka Kusunoki, Haruka Maruyama, Takuo Enomoto, Mutsutomo Tokizawa, Satoshi Iuchi, Masatomo Kobayashi, Leon V. Kochian, Hiroyuki Koyama, Yuriko Kobayashi

**Affiliations:** ^1^ Faculty of Applied Biological Sciences Gifu University Gifu Gifu Japan; ^2^ Global Institute for Food Security University of Saskatchewan Saskatoon SK Canada; ^3^ Experimental Plant Division RIKEN BioResource Research Center Tsukuba Ibaraki Japan

**Keywords:** Al tolerance, GWAS, MATE, natural variation, STOP1, transposon

## Abstract

Organic acids (OA) are released from roots in response to aluminum (Al), conferring an Al tolerance to plants that is regulated by OA transporters such as ALMT (Al‐activated malate transporter) and multi‐drug and toxic compound extrusion (MATE). We have previously reported that the expression level polymorphism (ELP) of *AtALMT1* is strongly associated with variation in Al tolerance among natural accessions of Arabidopsis. However, although *AtMATE* is also expressed following Al exposure and contributes to Al tolerance, whether *AtMATE* contributes to the variation of Al tolerance and the molecular mechanisms of ELP remains unclear. Here, we dissected the natural variation in *AtMATE* expression level in response to Al at the root using diverse natural accessions of Arabidopsis. Phylogenetic analysis revealed that more than half of accessions belonging to the Central Asia (CA) population show markedly low *AtMATE* expression levels, while the majority of European populations show high expression levels. The accessions of the CA population with low *AtMATE* expression also show significantly weakened Al tolerance. A single‐population genome‐wide association study (GWAS) of *AtMATE* expression in the CA population identified a retrotransposon insertion in the *AtMATE* promoter region associated with low gene expression levels. This may affect the transcriptional regulation of *AtMATE* by disrupting the effect of a cis‐regulatory element located upstream of the insertion site, which includes AtSTOP1 (sensitive to proton rhizotoxicity 1) transcription factor‐binding sites revealed by chromatin immunoprecipitation‐qPCR analysis. Furthermore, the GWAS performed without the accessions expressing low levels of *AtMATE*, excluding the effect of *AtMATE* promoter polymorphism, identified several candidate genes potentially associated with *AtMATE* expression.

## 1 INTRODUCTION

Aluminum (Al) stress caused by the rhizotoxicity of Al ions in acid soil solution is one of the most serious environmental stress factors disturbing plant growth in arable land worldwide (see review, Kochian et al., 2004). The rhizotoxicity of Al is caused by its cyto‐ and geno‐toxicities that disturb both cell elongation and division of the root apex (Ma, 2007). Al disturbs various biological processes of the root tip cells and subsequently induces reactive oxygen species (ROS)‐mediated damage (Yamamoto et al., 2002; Ma, 2007). Taken together, Al severely inhibits root growth, making plants very susceptible to drought stress (Ma, 2005). In agriculture, the breeding of Al‐tolerant crop varieties is important because approximately 40% of the global arable land is acidic (von Uexküll and Mutert, 1995). The acid soils occur naturally in high‐rainfall regions, where alkaline salts in soils are breached out by the naturally occurring acidic rainwater saturated with carbon dioxide (Krug and Frink, 1983). This suggests that the natural variation in Al tolerance occurs depending on the soil pH of the geographical location of the vegetation. Actually, Al‐tolerant Arabidopsis accessions were found in acid soil areas (Nakano et al., 2020). Therefore, elucidating the alteration mechanisms of Al tolerance among natural vegetation would lead to better understanding of the molecular mechanisms underlying the generation of variation in acid soil tolerance among intraspecific accessions.

Plants have evolved several Al tolerance mechanisms for adaptation to acid soil environment. The secretion of root organic acid (OA), which chelates toxic Al ions from the rhizosphere, is one of the most important Al tolerance mechanisms (Kochian et al., 2004), and this mechanism is substantially conserved across plant species (Ma et al., 2001). The OA transporters playing a key role in Al tolerance are divided in two families, ALMT (Al‐activated malate transporter) and multi‐drug and toxic compound extrusion (MATE), in plants. *ALMT* and *MATE* have been identified as major Al tolerance genes in wheat (*Triticum aestivum* L.)/Arabidopsis and sorghum (*Sorghum bicolor*)/maize (*Zea mays*)/barley (*Hordeum vulgare* L.), respectively (Sasaki et al., 2004; Hoekenga et al., 2006; Furukawa et al., 2007; Magalhaes et al., 2007; Maron et al., 2010). However, in addition to the primary OA transporter, a secondary transporter contributes to Al tolerance. Usually, citrate ions have a higher Al‐chelating capacity than malate ions (Li et al., 2000); therefore, a citrate release along with a malate release, could contribute to the effective detoxification of Al in the rhizosphere of the plants primarily releasing malate ions from their roots. Arabidopsis releases a small amount of citrate via AtMATE, a functional homologue of SbMATE, in response to Al stress, coincident with the release of a large amount of malate via AtALMT1, and it has been reported that the release of citrate ions acts as a secondary Al tolerance mechanisms in Arabidopsis (Liu et al., 2009; Liu et al., 2012). Similarly, in wheat plants, which mainly release malate via TaALMT1, citrate release via TaMATE1 also contributes to Al tolerance in addition to malate release in a citrate‐efflux genotype (Ryan et al., 2009). Accordingly, a secondary OA transporter might also contribute to Al tolerance in each plant species in addition to the primary contributor.

The genes encoding OA transporters involved in Al tolerance show high expression under Al stress conditions (Kobayashi et al., 2007; Magalhaes et al., 2007; Liu et al., 2009; Maron et al., 2010). Previous reports demonstrated that the quantitative difference in the gene expression levels (i.e., expression level polymorphism [ELP]) of an OA transporter correlated with Al tolerance among cultivars in various crops (Sasaki et al., 2006; Magalhaes et al., 2007; Fujii et al., 2012; Chen et al., 2013; Yokosho et al., 2016; Kashino‐Fujii et al., 2018). The ELP of an OA transporter was also associated with the soil pH of the cultivated area of the cultivars, suggesting that ELP drives the adaptation to acid soil environment in regions where higher Al tolerance is required (Fujii et al., 2012). Taken together, the ELP of an OA transporter is an important determinant for generating natural variation in Al tolerance. However, there are only a few reports regarding the ELP of a secondary OA transporter (e.g., *AtMATE*) and the contribution of ELP in Al tolerance variation. Beside, in Arabidopsis, *AtMATE* gene expression is regulated by AtSTOP1 (sensitive to proton rhizotoxicity 1) and AtSTOP2 as well as *AtALMT1* (Iuchi et al., 2007; Liu et al., 2009; Kobayashi et al., 2014). Similarly, in other plant species, the genes encoding *STOP1*‐like transcription factors (TFs) regulate *MATE* gene expression (Yamaji et al., 2009; Ohyama et al., 2013; Sawaki et al., 2014; Fan et al., 2015; Huang et al., 2018). Recently, we found that the PtdIns‐4‐kinase (PI4K) pathway regulated the AtSTOP1‐regulating genes of *AtALMT1*, *AtMATE*, and *ALS3* (Wu et al. 2019). However, the mechanism of transcriptional regulation of *AtMATE* and its association with natural variation are mostly unknown.

Recent advances in next‐generation sequencing technologies have allowed calling high‐density single nucleotide polymorphism (SNP) markers across numerous accessions (The 1001 Genomes Consortium, 2016), facilitating high‐resolution genome‐wide association study (GWAS). In contrast to biparental quantitative trait loci (QTL) mapping, GWAS can explore the genetic factor underlying the natural variation from a diverse genetic pool of multiple accessions. The genetic factors determining the ELP of several stress tolerance genes have been identified by association mapping using a diversity panel; these factors are known to drive adaptation to environments (Baxter et al., 2010; Yang et al., 2013). Recently, we successfully identified the mechanisms underlying the ELP of the genes associated with H_2_O_2_ and Al tolerance by expression GWAS (eGWAS) using Arabidopsis accessions (Sadhukhan et al., 2017; Nakano et al., 2020). In the current study, we analyzed the ELP of *AtMATE* among Arabidopsis natural accessions to evaluate the contribution of a secondary OA transporter in Al tolerance variation and to identify the genetic factors involved in the transcriptional regulation of *AtMATE*. We found that some of the accessions belonging to the Central Asia (CA) population showed markedly lower *AtMATE* expression levels and significantly lower Al tolerance compared to the other accessions. Furthermore, we performed a single‐population eGWAS using only the CA population, which successfully identified the promoter polymorphism of *AtMATE* resulting in markedly lower *AtMATE* expression levels.

## 2 MATERIAL AND METHODS

### 2.1 Plant material

Fifty Arabidopsis accessions used in this study were obtained from the Arabidopsis Biological Resource Centre (ABRC), Nottingham Arabidopsis Stock Centre (NASC), and RIKEN BioResource Research Center (RIKEN BRC). The progeny seeds of the accessions derived via the single‐seed descent method from the obtained seeds were used for the experiments (Table S1).

### 2.2 RNA extraction and measuring gene expression level

Hydroponic culture and Al stress treatment were conducted as described previously (Kobayashi et al., 2007). Approximately 100 seedlings of each accession were grown in modified 2% MGRL nutrient solution (Fujiwara et al., 1992) (pH 5.6) for 10 days. The nutrient solution was renewed every 2 days. Subsequently, the seedlings were transferred to the Al stress solution (modified 2% MGRL without Pi, 10 μM AlCl_3_, pH 5.0) according to a previously described method (Kusunoki et al., 2017). After incubation for 9 hr under Al stress conditions, the roots of the seedlings were harvested and immediately frozen in liquid nitrogen. Total RNA extraction and reverse transcription were performed using Sepasol‐RNA I Super G (Nacalai Tasque) and ReverTra Ace (Toyobo) in accordance with the manufacturer’s instructions. The gene expression levels of the accessions were measured by a quantitative real‐time polymerase chain reaction (qRT‐PCR) with THUNDERBIRD SYBR qPCR Mix (Toyobo) and Thermal Cycler Dice Real Time System II (Takara Bio) according to the manufacturer’s instructions using gene‐specific primer pairs (Table S2). The expression level of *AtSAND* (At2g28390) was used as an internal control. The expression level of the *AtMATE* (At1g51340) of each accession was calculated as the mean of three replicates (one replicate consists of 100 seedlings), and normalized by the expression level of Col‐0 as the control for experimental batches.

### 2.3 Phylogenetic analysis and GWAS

The SNP information obtained from the 1001 genomes project was used for the phylogenetic analysis and GWAS performed in this study (Cao et al., 2011; Horton et al., 2012; The 1001 Genomes Consortium, 2016). A phylogenetic tree of 50 accessions was constructed by the neighbor‐joining method using 164 842 genome‐wide SNPs (minor allele frequency [MAF] > 5%, missing call < 10%) in MEGA 6.0 (Tamura et al., 2013). The information of the geographic origin of each accession was obtained from the AraPheno database (https://arapheno.1001genomes.org; Seren et al., 2017), and the mapping of accessions onto a world map was performed by the “Geocoding and Mapping” web tool (http://ktgis.net/gcode/lonlatmapping.html).

The GWAS for the *AtMATE* expression level of the CA population and higher‐expression group was performed with the general and mixed linear models (GLM and MLM), respectively, using the 164 842 genome‐wide SNPs in TASSEL 5.0 (Bradbury et al., 2007). The information of the position and description of the Arabidopsis genes located around the GWAS‐detected SNPs was obtained from the Araport11 database (http://www.araport.org/; Cheng et al., 2017). The information of the Al tolerance levels of the accessions belonging to the CA population was obtained from Nakano et al., (2020).

### 2.4 PCR amplification and sequence analysis of the *AtMATE* promoter region

The *AtMATE* promoter regions of the CA accessions were amplified by KOD FX Neo (Toyobo) using a primer pair to amplify approximately 3 kb of the upstream region of *AtMATE* (Table S2). The long insertion in the *AtMATE* promoter region amplified from genomic DNA (gDNA) of Sij‐4 (lower‐expression type accession) was sequenced by next‐generation sequencing after gel extraction using NucleoSpin Gel and PCR Clean‐up (MACHEREY‐NAGEL) according to the manufacturer’s instructions. Library preparation was carried out by using the Nextera XT DNA Library Preparation Kit (Illumina). Sequencing was conducted by using the Illumina MiSeq System in a paired‐end mode with a read length of 300 bp. In order to maintain the higher quality of reads, a 120‐bp fragment of the 3′‐end of each read was trimmed, and then the removal of the sequencing primer sequence and quality filtering were performed using Trimmomatic 0.38 (Bolger et al., 2014). The filtered reads were used for de novo assembly by Velvet v. 1.2.10 and VelvetOptimiser v. 2.2.6 (Zerbino and Birney, 2008). Both ends of the insertion that did not assemble were sequenced by the ABI PRISM 3130xl DNA sequencer with the BigDye Terminator v3.1 Cycle Sequencing Kit (Thermo Fisher Scientific), using a PCR product amplified from the gDNA of Sij‐4 by TaKaRa Ex Taq Hot Start Version (Takara Bio) according to the manufacturer’s instructions. The primers are described in Table S2.

### 2.5 Promoter GUS assay in hairy root

The *AtMATE promoter*::GUS fusion constructs were generated by performing an overlap extension PCR (Horton et al., 1989) using the PrimeSTAR Max DNA polymerase (Takara Bio) and then introduced into the binary vector pBE2113. The primers used for this assay are described in Table S2. The vectors carrying the constructs were introduced into *Agrobacterium rhizogenes* ATCC15834 by electroporation. Transgenic hairy roots were generated from the Arabidopsis stem according to the method described in Daspute et al. (2018) with a minor modification. *Agrobacterium rhizogenes* carrying each vector were infected into the chopped Arabidopsis stems by dipping, and then the stems were cultivated in half‐strength MS medium (Murashige and Skoog, 1962) with sucrose (1%, w/v), agar (1%, w/v), and acetosyringone (0.2 mM). After 3 days of cultivation, the stems were transferred to the selection medium (half‐strength MS medium containing 1% sucrose, 1% agar, 3.11 mg/L meropenem, 1.0 ml/L 1000x Gamborg’s Vitamin Solution [Sigma‐Aldrich, St. Louis, MO, USA], 100 mg/L cefotaxime, and 10 mg/L hygromycin B). The selection medium was renewed every 1 week until hairy root formation. The formed hairy roots were used for measuring GUS expression. Approximately 20–30 hairy roots were transferred to Al stress hydroponic solution (modified 2% MGRL without Pi, 10 μM AlCl_3_, pH 5.0). After incubation for 9 hr, the hairy roots were immediately frozen in liquid nitrogen. Total RNA extraction and reverse transcription were performed using the RNeasy Plant Mini Kit (QIAGEN) and ReverTra Ace (Toyobo) in accordance with the manufacturer’s instructions. The gene expression levels were measured using gene‐specific primer pairs (Table S2) as described above. The expression level of *HPT*, a gene in the plasmid vector, was used as an internal control to normalize the site of transgene insertion as described by Tovkach et al. (2013).

### 2.6 Chromatin immunoprecipitation

Chromatin immunoprecipitation (ChIP) was conducted using the method described previously (Gendrel et al., 2005; Ogita et al., 2018). The Col‐0 and transgenic Arabidopsis plants expressing *AtSTOP1* promoter::AtSTOP1‐GFP were grown in 2% MGRL solutions. *AtSTOP1* promoter::AtSTOP1‐GFP transgenic was generated as described in Ohyama et al. (2013). The PCR fragment of from *AtSTOP1* promoter to coding region was connected to PCR fragment combining sGFP and nos terminator by overlap extension PCR (Horton et al., 1989). The fragment was inserted into the binary vector pBE2113. These primers in the PCR are described in Table S2. The 10‐day‐old seedlings were treated with or without 10 µM AlCl_3_ for 6 hr. Approximately 120 mg of roots (approximately 800–1,000 plants) were collected, and the cross‐linking reaction was performed with 1% formaldehyde (Sigma‐Aldrich) under vacuum conditions. The chromatin DNA was fragmented using the sonic dismembrator M120 (Fisher Scientific) under the following conditions: 50% amplitude and 23 cycles of 10 s “ON”/2 min “OFF”. An anti‐GFP polyclonal antibody (A6455, Invitrogen) and Dynabeads™ Protein G (Invitrogen) were used for immunoprecipitation assay. To qualify immunoprecipitated DNA, a qPCR assay was conducted using the Quant Studio 6 Real‐Time PCR System and Power Up SYBR Green Master Mix (Applied Biosystems). The primers used for the qPCR assay are shown in Table S2. The primers for the mutator‐like transposon (Mut‐like) were used as a negative control (Sauret‐Güeto et al., 2013). Two independent ChIP assays were conducted, and similar results were obtained.

### 2.7 Accession numbers

The *AtMATE* promoter sequences of Sij‐4 and Shigu‐1 determined in this study were submitted to the DDBJ database under the accession numbers LC534896 and LC534897, respectively.

## 3 RESULTS

### 3.1 ELP of *AtMATE*

**FIGURE 1:**
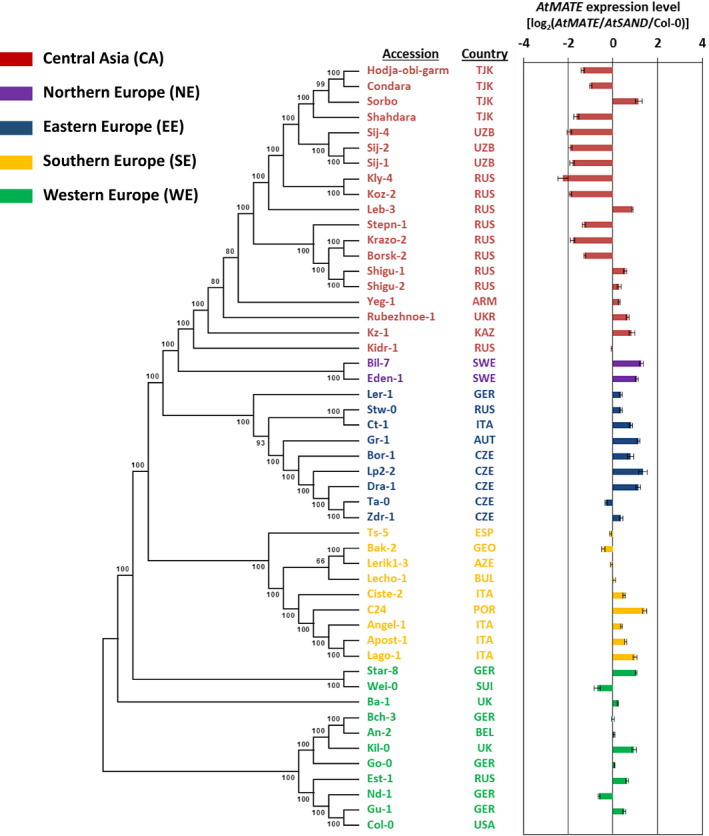
Phylogenetic tree of the accessions constructed by the neighbor‐joining method using 164 842 genome‐wide single nucleotide polymorphisms (SNPs). The bootstrap values (100 replicates) are shown next to the branches. The information of the country‐of‐origin of each accession was obtained from the Araport11 database (Cheng et al., 2017). The colors correspond to the subpopulations that were classified based on the phylogenetic tree and their geographic origin. *AtMATE* expression levels are represented as mean ± SD (*n* = 3)

We measured the *AtMATE* expression levels of 50 Arabidopsis accessions collected by the 1001 Genomes Project (Weigel and Mott, 2009; Cao et al., 2011; Horton et al., 2012) (Table S1). Approximately 100 seedlings of each accession were grown hydroponically for 10 days under control conditions and then treated with Al for 9 hr. Subsequently, total RNA was isolated from the roots, and the *AtMATE* expression levels were determined by qPCR. The segregation of the *AtMATE* expression levels was a bimodal distribution with the two different groups of low‐level expression (−2.2 to −1.0) and high‐level expression (−0.71 to 1.42) (Figure S1). We constructed a phylogenetic tree of the 50 accessions using 164 842 genome‐wide SNPs and analyzed the relationships between the *AtMATE* expression levels and genetic backgrounds of the studied accessions (Figure 1). Based on the results of phylogenetic tree, the accessions were divided into five subpopulation, which were associated with their geographic origin obtained from AraPheno (Cheng et al., 2017); each subpopulation roughly corresponded to the geographic regions of CA, Northern Europe (NE), East Europe (EE), Southern Europe (SE), and Western Europe (WE), respectively (Figure S2A). This analysis revealed that more than half of the accessions belonging to the CA population showed markedly lower *AtMATE* expression level compared to other accessions belonging to CA and other subpopulations (Figure 1). Thus, the markedly lower‐expression group in the histogram was composed of only the accessions belonging to the CA population, while the higher‐expression group consisted of the accessions belonging to various populations (Figure S1). These results suggested that a genetic factor with a large effect determining the markedly lower expression level of *AtMATE* was shared within accessions of the CA population.

### 3.2 Single‐population GWAS for *AtMATE* expression level

**FIGURE 2:**
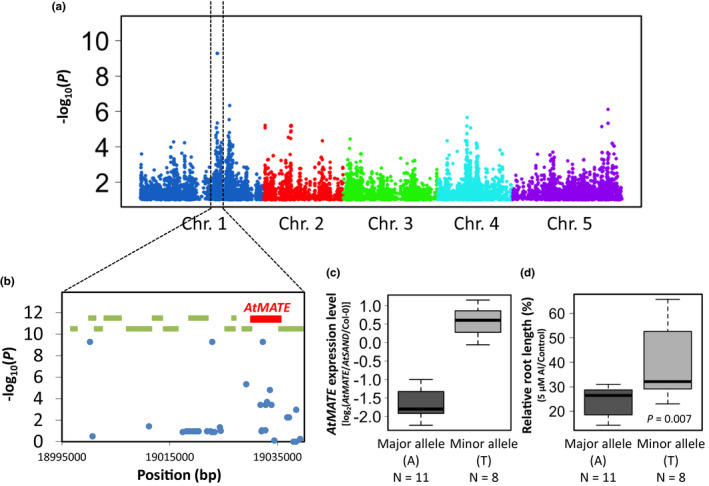
Single‐population genome‐wide association study (GWAS) for *AtMATE* expression level. (a) A Manhattan plot of GWAS for *AtMATE* expression level using 19 accessions belonging to the Central Asia (CA) population. Only single nucleotide polymorphisms (SNPs) with a *p*‐value < .1 are shown. (b) Detailed plots of the most associated SNPs on Chr.1. Green rectangles represent the genes located in the region, and red rectangle indicates *AtMATE*. (c and d) Boxplots of the *AtMATE* expression level and relative root length of the higher associated SNP in the *AtMATE* locus (Chr.1_19032290). The *p*‐value for the relative root length was calculated by the Student’s *t* test

To identify the genetic factor determining the markedly lower expression level of *AtMATE*, we performed a GWAS based on the *AtMATE* expression level using only the CA population containing 19 accessions (Single‐population GWAS) to try to reduce the effect of population structure and genetic heterogeneity caused by high genetic diversity across all accessions. As a result of the GWAS, three most strongly associated SNPs were detected on chromosome 1 (*p* = 5.2 × 10^−10^) (Figure 2A), with two of the SNPs located in the intragenic region and another in the coding region of *AtMATE* (Chr1_19032290; Figure 2B). Of the 19 accessions used for the GWAS, 11 carried a major allele (A) of the associated SNP located in the *AtMATE* gene, and they showed approximately one‐fifth of the mean *AtMATE* expression level than those carrying the minor allele (T) (Figure 2C). Additionally, the accessions carrying the major allele showed significantly lower Al tolerance, which was evaluated by the relative root length (root length under Al‐positive conditions/root length under Al‐negative conditions) as an index, than in the accessions carrying the minor allele (Figure 2D). This result suggested that the markedly lower *AtMATE* expression level was caused by the polymorphism of the *AtMATE* locus, and it contributed to the generation of Al tolerance variation among Arabidopsis accessions.

### 3.3 *AtMATE* promoter polymorphism associated with the expression level

**FIGURE 3:**
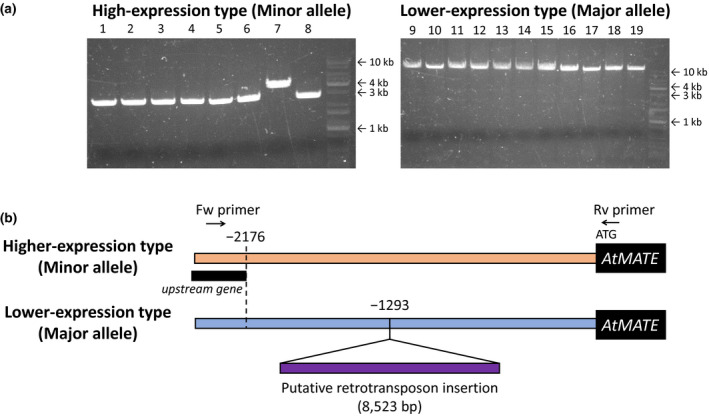
Polymorphism of *AtMATE* promoter among Arabidopsis accessions. (a) Polymerase chain reaction (PCR) amplification analysis of *AtMATE* promoter region for higher‐expression‐type accessions (1: Kz‐1, 2: Shigu‐1, 3: Sorbo, 4: Yeg‐1, 5: Shigu‐2, 6: Rubezhnoe‐1, 7: Kidr‐1, 8: Leb‐3) and lower‐expression‐type accessions (9: Sij‐2, 10: Stepn‐1, 11: Sij‐1, 12: Koz‐2, 13: Shahdara, 14: Condara, 15: Hodja‐obi‐garm, 16: Borsk‐2, 17: Kly‐4, 18: Sij‐4, 19: Krazo‐2,). (b) Schematic representation of the higher‐ and lower‐expression‐type *AtMATE* promoters. A retrotransposon insertion of 8523 bp was detected at −1,293 bp upstream of the start codon in the lower‐expression‐type promoter (Sij‐4). Black rectangle represents the upstream neighboring gene (At1g51330) of *AtMATE*. The Black arrows indicate the primers used for PCR amplification on (a)

Because the single‐population GWAS for *AtMATE* expression level detected a prominent association of the *AtMATE* locus, we tried to identify the causal polymorphism determining the variation of *AtMATE* expression level. First, we compared approximately 3 kb sequence from the start codon of the *AtMATE* gene to the next gene, based on the sequence of Col‐0 obtained from the Arabidopsis Information Resource (TAIR) database, as a potential promoter region between the accessions with minor and major alleles of the GWAS‐detected SNPs in the locus. The PCR amplification analysis of the promoter region amplified approximately 3–4 kb product from the eight accessions with the minor allele, while approximately 10 kb products were obtained from the 11 accessions with the major allele (Figure 3A). This result suggested that a long insertion existed in the *AtMATE* promoter region of the accessions with the major allele, and it is completely linked with the GWAS‐detected SNPs associated with the *AtMATE* expression level variation in the CA population. To characterize the long‐insertion, we conducted amplicon sequencing for the insertion amplified from the Sij‐4 (low‐expression type) gDNA using the Illumina Miseq System. This analysis revealed an 8523‐bp insertion located 1,293 bp upstream of the start codon (Figure 3B). This insertion was flanked by a 5‐bp target site duplication (AAGTG), and approximately 5‐kb of the sequence was matched for the transposable element gene (At1g51175; gypsy‐like retrotransposon family) located approximately 60 kb upstream of the *AtMATE* gene (At1g51340) in Col‐0 (no insertion, high‐expression type), suggesting that the long insertion in the promoter region was derived from the retrotransposon. These results indicated that the retrotransposon insertion into the promoter region is the most possible causal variant determining the markedly lower *AtMATE* expression level.

### 3.4 Promoter analysis of *AtMATE* associated with the expression level

**FIGURE 4:**
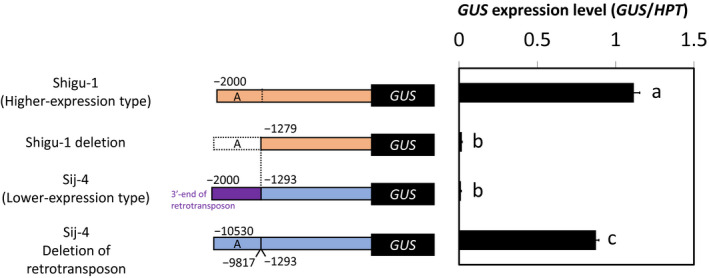
Promoter activity analysis of the *AtMATE* promoters. Higher‐ (Shigu‐1) and lower‐expression‐type (Sij‐4) promoters are indicated by orange and blue boxes, respectively. Retrotransposon sequence is indicated by a purple box. Dotted rectangle indicates deletion region. Homologous sequences are marked with an "A" to each other. Approximately 20–30 transgenic hairy roots were incubated in the Al stress solution (10 µM Al, pH 5.0) for 9 hr. The *GUS* expression levels were measured by a quantitative real‐time polymerase chain reaction (qPCR). The expression level of *HPT* was used as an internal control. Different letters indicate significant differences (Tukey’s HSD test; *p* < .05). Mean values ± SD are shown (*n* = 3)

To evaluate the effect of retrotransposon insertion on *AtMATE* expression level, we compared the activity of the *AtMATE* promoters of the higher‐expression‐type (Shigu‐1) and lower‐expression‐type (Sij‐4) accessions by performing a promoter GUS assay in the hairy root of Arabidopsis (Figure 4). The 2 kb of Sij‐4 promoter, containing the approximately 700 bp of 3′‐end of the 8.5 kb transposon, provided very low *GUS* expression level compared to that of the Shigu‐1 promoter. In contrast, the *GUS* expression level of 2 kb of transposon‐less Sij‐4 promoter was increased to approximately that of higher‐expression‐type promoter (i.e., Shigu‐1 promoter). In addition, the Shigu‐1 promoter lacking approximately 700 bp upstream sequence from the transposon insertion sites in Sij‐4, whose sequence corresponded to the −9,817 to –10,530 region upstream of the transposon in Sij‐4 (as shown by “A” fragment in Figure 4), provided very low *GUS* expression level in the manner similar to the Sij‐4 promoter. These results indicated that the upstream promoter region of the transposon insertion plays an important role in the transcriptional regulation of *AtMATE*.

**FIGURE 5:**
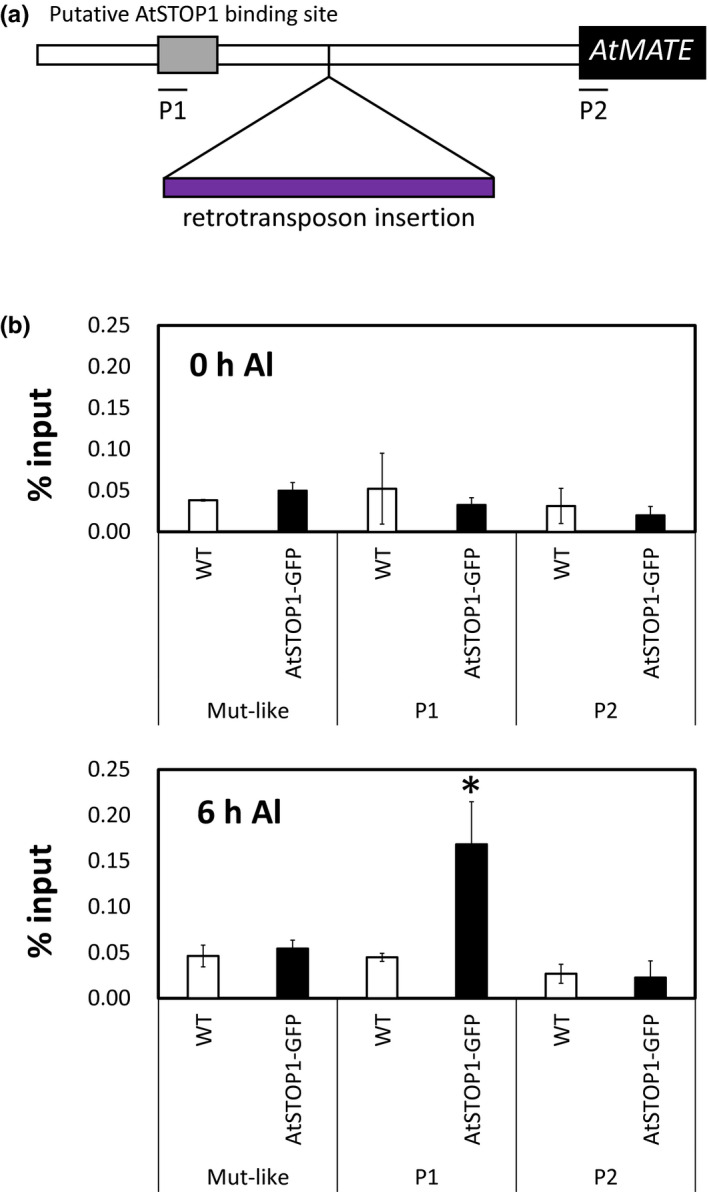
Chromatin immunoprecipitation quantitative real‐time polymerase chain reaction (ChIP‐qPCR) of AtSTOP1 to the *AtMATE* promoter. (a) Location of the putative AtSTOP1‐binding sites on the *AtMATE* promoter. Black box indicates the putative AtSTOP1‐binding region inferred from the Plant Cistrome Database. P1 and P2 represent the amplified regions in ChIP‐qPCR. (b) ChIP‐qPCR of the upstream promoter region of *AtMATE*. The ChIP fragments were obtained with anti‐GFP polyclonal antibody from WT and transgenic plant expressing *AtSTOP1*promoter::AtSTOP1‐GFP after 0 hr or 6 hr Al treatment. The enrichment of qPCR products from immunoprecipitated DNA are normalized by input DNA. Mutator‐like transposon (Mut‐like) was measured as the negative control. Mean values ± SD are shown (*n* = 3). Asterisk indicates the significant difference from the WT (Student’s *t* test; *p* < .05)

Next, to characterize the role of the region (as shown by “A” fragment in Figure 4) in *AtMATE* expression, we identified a series of TFs binding to the region (Chr1_19029996‐19030722 in Col‐0) using the DNA affinity purification sequencing (DAP‐seq) data obtained from the Plant Cistrome Database (http://neomorph.salk.edu/dap_web/pages/index.php; O’Malley et al., 2016). This analysis identified a total of 22 TFs potentially binding to the upstream region (Table S3); these TFs included AtSTOP1, which is known to be important for *AtMATE* transcriptional regulation (Liu et al., 2009). Two Dap‐seq peaks of AtSTOP1 were detected closely each other on the upstream region (−1,603 and −1,496 bp away from the start codon, respectively; Figure 5A). In fact, this region contained several minimum consensus sequences of ART1/STOP1 (GGNVS; Tsutsui et al., 2011; Tokizawa et al., 2015), suggesting that AtSTOP1 directly regulates the expression of *AtMATE* by binding to this region. Furthermore, to confirm whether AtSTOP1 binds to the promoter region under Al stress conditions, we conducted a ChIP‐qPCR analysis using a AtSTOP1‐GFP transgenic plant. The analysis detected significant AtSTOP1 binding for the promoter region at 6 hr of Al treatment, while no significant level of AtSTOP1 binding was detected at 0 hr Al treatment (Figure 5B). This result indicated that AtSTOP1 binds to the promoter region of *AtMATE* and regulates its expression under Al stress conditions.

### 3.5 Association mapping for *AtMATE* expression level in the higher‐expression group

We found retrotransposon insertion as the causal variant determining the markedly low *AtMATE* expression level of the 11 accessions in the CA population from the single‐population GWAS (Figure 2). However, a variation in *AtMATE* expression level was still observed within the higher‐expression group, except the 11 accessions with low *AtMATE* expression level (Figure S1), suggesting the existence of other genetic factors to explain the variation of the group, which might be common across multiple subpopulations. Next, we attempted to map the loci associated with the ELP of the higher‐expression group by GWAS.

**FIGURE 6:**
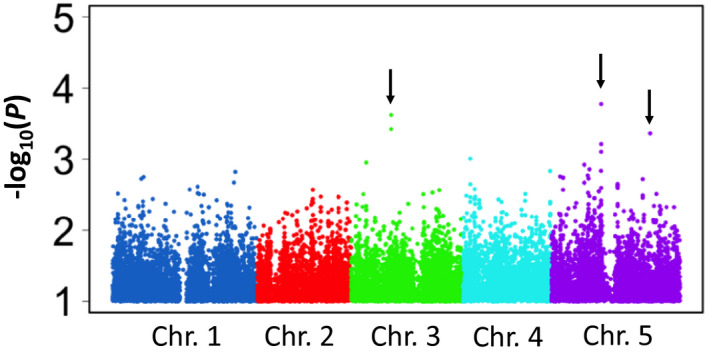
Manhattan plot of the genome‐wide association study (GWAS) for the *AtMATE* expression level in the higher‐expression group. Only single nucleotide polymorphisms (SNPs) with a *p*‐value < .1 are shown. Black allows indicate the highlighted‐SNPs (Chr.3_8382376, Chr.5_10425150, and Chr.5_20724966)

The GWAS for the *AtMATE* expression levels was conducted using 39 accessions constituting a higher‐expression group without the low‐expression 11 accessions. We highlighted the three strongest associated loci at one locus on chromosome 3 and two loci on chromosome 5 (Figure 6). The most associated SNPs on these loci were Chr.3_8382376 (*p* = 2.4 × 10^−4^), Chr.5_10425150 (*p* = 1.7 × 10^−4^), and Chr.5_20724966 (*p* = 4.4 × 10^−4^). These were different from the *AtMATE* loci detected in the above‐mentioned single‐population GWAS. These results suggested that some trans‐regulatory factors are involved in the ELP of *AtMATE* expression level in addition to the cis‐regulatory factor. Several genes are located within a span of ±10 kb, which is the average linkage disequilibrium decay distance of Arabidopsis (Kim et al., 2007), from the SNPs (Table 1). The gene list contained several potential candidates that could regulate *AtMATE* expression. For example, *AtPHO1* (*phosphate 1*), which is involved in cellular response to phosphate starvation and inositol phosphate‐mediated signaling, and *AtFUM2* (*fumarase 2*), which is involved in tricarboxylic acid cycle. Further characterization for these collocated genes will lead to the identification of the genes determining natural variation in *AtMATE* expression among higher‐expression accessions.

**TABLE 1:** List of genes located ±10 kb from the genome‐wide association study (GWAS)‐detected single nucleotide polymorphisms (SNPs)

Chr_SNP	AGI code	Description
3_8382376	AT3G23380	ROP‐interactive CRIB motif‐containing protein 5 (RIC5)
	AT3G23390	Zinc‐binding ribosomal protein family protein
	AT3G23400	Fbrillin family protein fibrillin 4 (FIB4)
	AT3G23410	Fatty alcohol oxidase 3 (FAO3)
	AT3G23420	F‐box and associated interaction domains‐containing protein
	AT3G23430	Phosphate 1 (PHO1)
5_10425150	AT5G04755	Long noncoding RNA
	AT5G28470	Major facilitator superfamily protein
5_20724966	AT5G50920	CLPC homologue 1 (CLPC1)
	AT5G50930	Histone superfamily protein
	AT5G50940	RNA‐binding KH domain‐containing protein
	AT5G50950	FUMARASE 2 (FUM2)
	AT5G50960	Nucleotide‐binding protein 35 (NBP35)
	AT5G50970	Transducin family protein/WD‐40 repeat family protein

## 4 DISCUSSION

In this study, we identified a retrotransposon insertion into the *AtMATE* promoter region as the causal genetic factor determining markedly lower *AtMATE* expression level of 11 CA accessions by performing a single‐population GWAS. Furthermore, we found that the upstream promoter region of the retrotransposon insertion site contributed largely to the *AtMATE* expression level, which might be involved in AtSTOP1‐mediated transcriptional regulation. This variant was mainly shared within the CA population and it contributed to the generation of natural variation in Al tolerance. In addition to the insertion, the GWAS using a higher‐expression group detected several potential candidates as trans‐acting factors, which might also influence the *AtMATE* expression level.

The ELP of a primary OA transporter is an important determinant of Al tolerance variation among intraspecific accessions (Hoekenga et al., 2006; Sasaki et al., 2006; Magalhaes et al., 2007). In Arabidopsis, it has been reported that the ELP of *AtALMT1* is associated with Al tolerance level among natural accessions (Hoekenga et al., 2006, Nakano et al., 2020). Besides, the Al‐inducible *AtMATE* expression secondly contributes to Al tolerance in a manner similar to *AtALMT1* (Liu et al., 2009). In this study, we found that 11 accessions belonging to the CA population showed markedly lower *AtMATE* expression level (Figure 1 and Figure S1), and lower Al tolerance level compared to the other accessions in the CA population (Figure 2C,D). These observations indicated that the ELP of a secondary OA transporter, as well as that of primary OA transporter, contributes to the generation of Al tolerance variation among intraspecific accessions. This hypothesis is supported by the observation that the Brazilian wheat cultivars carrying higher‐expression type *TaMATE1b* (secondary OA transporter in wheat) tend to show higher Al tolerance level than those carrying lower‐expression type OA transporters (Pereira et al., 2015). Interestingly, although the *AtALMT1* expression level of the CA accessions with lower *AtMATE* expression tend to lower than that of the CA accessions with higher *AtMATE* expression, several accessions with lower *AtMATE* expression showed higher *AtALMT1* expression level in spite of the weak Al tolerance level (Figure 2D and Figure S3). This suggested that the ELP of secondary transporter (i.e., *AtMATE*) might have large contribution to Al tolerance variation than that of primary OA transporter (i.e., *AtALMT1*) in the local population. Furthermore, all 19 CA accessions used in this study were distributed in the alkaline soil where Al toxicity might be lower (Figure S2B). These observations suggested that higher Al tolerance was not essential to survive in their natural habitat. However, although there is relatively low threat of Al rhizotoxicity, iron (Fe) deficiency is a serious problem affecting plant growth in alkaline soil. It is known that a citrate transporter plays an important role not only in Al tolerance, but also in Fe acquisition (Puig et al., 2007). Further investigation is needed to uncover the contribution of lower‐expression type *AtMATE* to adapt to alkaline soil environment.

In the histogram of *AtMATE* expression levels, the lower expression group was composed of the accessions belonging to only the CA population (Figure S1), suggesting that the genetic factor determining the markedly lower *AtMATE* expression level was shared within the CA population. To identify the genetic factor, we conducted a single‐population GWAS using only the CA population. In general, a single‐population GWAS can clearly detect the genetic factor underlying phenotypic variation within an any population by removing the effects of population structure and genetic heterogeneity, which could lead to false positive and negative associations in GWAS (Korte and Farlow, 2013; Imamura et al., 2016). Using this analysis, we successfully detected the prominently associated SNPs in the *AtMATE* locus (Figure 2A,B). A sequence analysis revealed that a retrotransposon insertion in the *AtMATE* promoter region is the most possible causal genetic factor determining the markedly lower *AtMATE* expression level (Figure 3). On the other hands, the GWAS using all 50 accessions also detected two strongly associated SNPs on *AtMATE* locus (Chr1_19029202 with *p* = 1.24 × 10^−4^ and Chr1_19033070 with *p* = 9.24 × 10^−5^) together with several associated SNPs located in other genomic regions (Figure S4 and Table S1). However, the SNPs in the *AtMATE* locus were not the same as that detected by the single‐population GWAS using CA accessions (Chr1_19032290) and not linked with the retrotransposon insertion. In fact, seven accessions other than CA also possessed the lower‐expression allele even though the accessions does not have the transposon insertion (Figure S5 and Table S1). This incompletely linkage between the lead SNP and causal variant might affect the association analysis of GWAS when using all accessions. These results suggested that a single‐population GWAS could detect the subpopulation specific genetic factor more effectively than the GWAS with MLM using diverse accessions.

It is known that the insertion of a transposable element (TE) into a regulatory region drives the alteration of the gene expression level (Feschotte, 2008). Especially in the OA transporters contributing to Al tolerance, TE insertion is a well‐observed way to alter the gene expression level and Al tolerance (Sasaki et al., 2006; Fujii et al., 2012; Tovkach et al., 2013; Yokosho et al., 2016; Kashino‐Fujii et al., 2018; Pereira and Ryan, 2019). For example, the insertion of a miniature inverted transposable element (MITE) was found in the promoter region of *SbMATE* in sorghum, and the number of repeats of the MITE was correlated with the high expression level among sorghum lines (Magalhaes et al., 2007). Furthermore, other reports showed that the correlation between TE insertions in the promoter region of the OA transporter genes and their high expression levels (Sasaki et al., 2006; Yokosho et al., 2016; Kashino‐Fujii et al., 2018). Although the insertion of most TEs into OA transporters have enhanced the expression level and Al tolerance (Pereira and Ryan, 2019), the retrotransposon insertion in the *AtMATE* promoter is associated with the lower expression and Al susceptibility (Figure 2C,D). This observation demonstrated that the insertion of TE into the regulatory region of an OA transporter does not always result in enhancing the expression level and Al tolerance.

**FIGURE 7:**
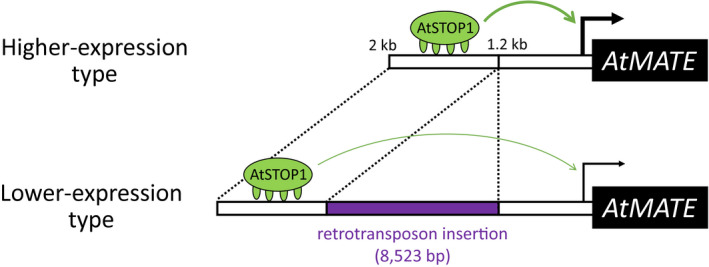
Hypothetical schematic of the effect of retrotransposon insertions on *AtMATE* gene expression levels. This is a comparison of higher‐expression type (accessions) 2‐kb promoter and lower‐expression type in which the transposon is inserted. AtSTOP1 binding site is much more upstream in the lower‐expression type than that of higher‐expression type. Purple region indicates transposon insertion in the lower‐expression type *AtAMTE* promoter. Green arrow indicates the effect of AtSTOP1 transcription factor for *AtMATE* transcription (black arrow)

Transposon insertion alters the expression of the flanking genes because of various kinds of effects, such as delivering a new cis‐acting element and changing DNA methylation pattern (Feschotte, 2008). The promoter *GUS* analysis revealed that the upstream promoter region of the retrotransposon insertion sites plays an important role in the transcriptional regulation of *AtMATE* (Figure 4). This result indicated that the retrotransposon insertion disrupted the *AtMATE* expression by increasing the distance between the cis‐elements located in the upstream promoter region and the transcription start site. Furthermore, we demonstrated that the upstream promoter region contained the AtSTOP1 binding sites (Figure 5). In *AtALMT1*, the expression level was greatly reduced by eliminating the AtSTOP1‐binding region of the promoter (Tokizawa et al., 2015). Consistent with this result, the promoter activity of *AtMATE* was greatly reduced by the deletion of the upstream region (Figure 4). These results suggested that retrotransposon insertion declined the *AtMATE* expression level by disrupting the AtSTOP1‐mediating transcriptional regulation (Figure 7). However, the accessions with the lower expression‐type *AtMATE* promoter showed a relatively high gene expression level compared to the *GUS* expression to the deletion promoter (Figures 2C and 4), suggesting that the cis‐elements in the upstream region still affect the transcriptional regulation of *AtMATE*. Further investigation is required to understand the interaction of promoter architecture and transcriptional regulation mediated by AtSTOP1.

In addition to the cis‐acting loci as being the retrotransposon insertion, we detected a few SNPs associated with the ELP of *AtMATE* by performing a GWAS using the higher‐expression group in the histogram of *AtMATE* expression levels, which was composed of the accessions belonging to various subpopulations, except for the 11 CA accessions carrying the retrotransposon insertion (Table 1). Although their association strength was lower than that of the *AtMATE* locus detected by the single‐population GWAS, several genes were expected to be involved in the *AtMATE* transcriptional regulation (Figure 6 and Table 1). The potential candidate genes included *AtPHO1*, which is involved in phosphate loading into the xylem, and a *pho1* mutant exhibits phosphate deficiency symptoms (Poirier et al., 1991; Hamburger, 2002). The cellular phosphate levels regulated by *AtPHO1* are triggered by the binding of inositol polyphosphate signaling molecules (Wild et al., 2016). Recently, we found that the phosphatidyl inositol metabolic pathway regulates the expression of *AtMATE* as well as *AtALMT1* under Al stress (Wu et al., 2019). Indeed, *LaMATE* is highly expressed not only under Al stress, but also under phosphate deficiency conditions in the proteoid roots of white lupin (*Lupinus albus*) (Uhde‐Stone et al., 2005). These reports suggest that *AtPHO1* might be involved in *AtMATE* expression via phospholipid signaling. Besides, other loci included one potential candidate gene of *AtFUM2*, whose ELP has been reported to be a determinant of the variation of fumarate/malate ratio among Arabidopsis accessions (Riewe et al., 2016). Arabidopsis has two fumarase genes, among which *AtFUM2* is involved in malate to fumarate interconversion in cytosol, and the knock‐out plant of *AtFUM2* showed significantly high citrate and malate content (Pracharoenwattana et al., 2010). The cellular OA content affect the amount of OAs secreted in response to Al stress. Several studies demonstrated that overexpressing mitochondrial citrate synthase resulted in an enhancement of the cellular citrate content, citrate release, and Al tolerance (Koyama et al., 1999; Koyama et al., 2000; Anoop, 2003). Furthermore, Wang et al. (2013) reported that the higher citrate or malate content resulting from the overexpression of mitochondrial citrate synthase lead to enhanced expression levels of OA transporters. These results suggested that the variation in OA content caused by the ELP of *AtFUM2* might be involved in the transcriptional regulation of *AtMATE*. However, further characterization of these potential candidate genes will be required to identify the genes underlying the regulation of the ELP of *AtMATE*, since there are still other possible candidates in the associated loci as shown in Table 1.

In conclusion, we found that the retrotransposon insertion into the *AtMATE* promoter is a major determinant of the ELP of *AtMATE*, and it has driven the variation of Al tolerance in a local population. Our result also indicated that the retrotransposon insertion affects the *AtMATE* expression level by disrupting the effect of cis‐elements located in upstream region of the insertion site, including the AtSTOP1‐binding sites.

## CONFLICT OF INTEREST

The authors declare no conflict of interest associated with the work described in this manuscript.

## AUTHOR CONTRIBUTIONS

Y.N., H.K., and Y.K. designed the research. Y.N., K.K., H.M., T.E., M.T., and S.I. performed research. Y.N., K.K., and M.T. analyzed data. Y.N., M.K., L.K., H.K., and Y.K. contributed new regents/analytic tools. Y.N., H.K., and Y.K. wrote the paper. All authors approved the manuscript.

## Supporting information

Fig S1‐S5‐Table S1‐S3
