# Supplementary material for: A single‐population GWAS identified AtMATE expression level polymorphism caused by promoter variants is associated with variation in aluminum tolerance in a local Arabidopsis population
Source: Plant Direct. 2020 Aug 12;4(8):e00250. doi: 10.1002/pld3.250 (PMC7419912; doi:10.1002/pld3.250)
Supplement: Supplementary file 1 — Fig S1‐S5‐Table S1‐S3 [file PLD3-4-e00250-s001.pdf]

Supplemental Figure S1

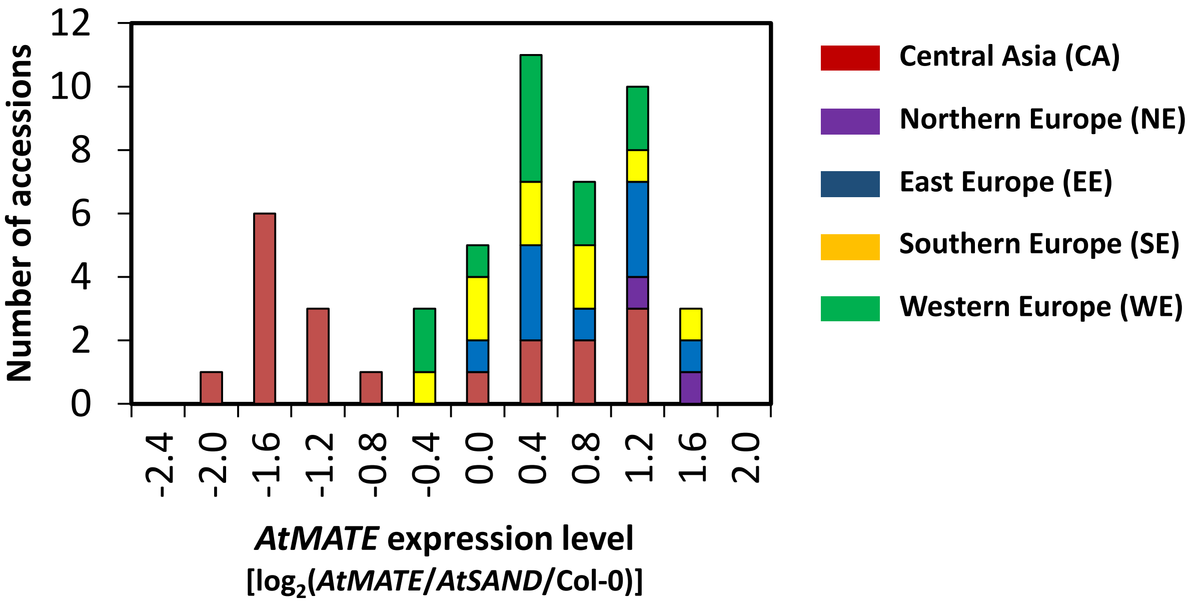

Supplemental Figure S1 A histogram of the *AtMATE* expression levels of the 50 accessions of *Arabidopsis thaliana*. Seedlings were grown hydroponically for 10 d in the control solution (0 µM Al, pH 5.6) and then transferred to the Al stress solution (10 µM, pH 5.0). After 9 h of incubation, the roots of the seedlings were harvested. Each color represents the subpopulation of accessions shown in Figure 1. The expression level of *AtSAND* was used as an internal control. The *AtMATE* expression level of Col-0 was used for the normalization of experimental batches.

Supplemental Figure S2

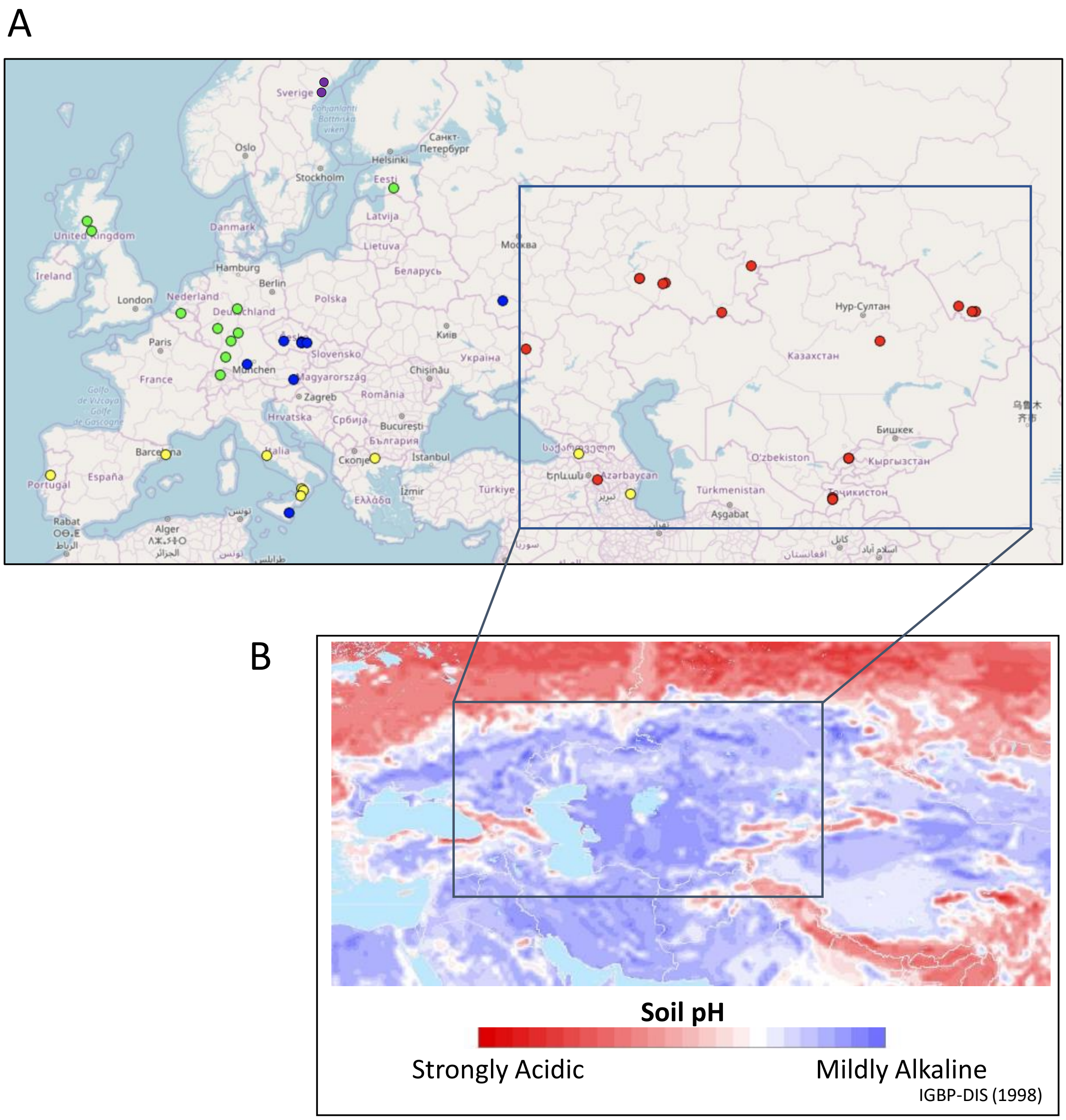

Supplemental Figure S2 Geographic distribution of the accessions used in this study. (A) Geographic origin of each accession. Each color corresponds to the five subpopulations shown in Figure 1 [red: Central Asia (CA); purple: Northern Europe (NE); blue: Eastern Europe (EE); yellow: Southern Europe (SE); green: Western Europe (WE)]. The information of the geographic origin of each accession was obtained from AraPheno (Seren et al., 2017). (B) Soil pH of the CA region (Source: IGBP-DIS (1998) SoilData (V.0) A program for creating global soil-property databases, IGBP Global Soils Data Task, France).

Supplemental Figure S3

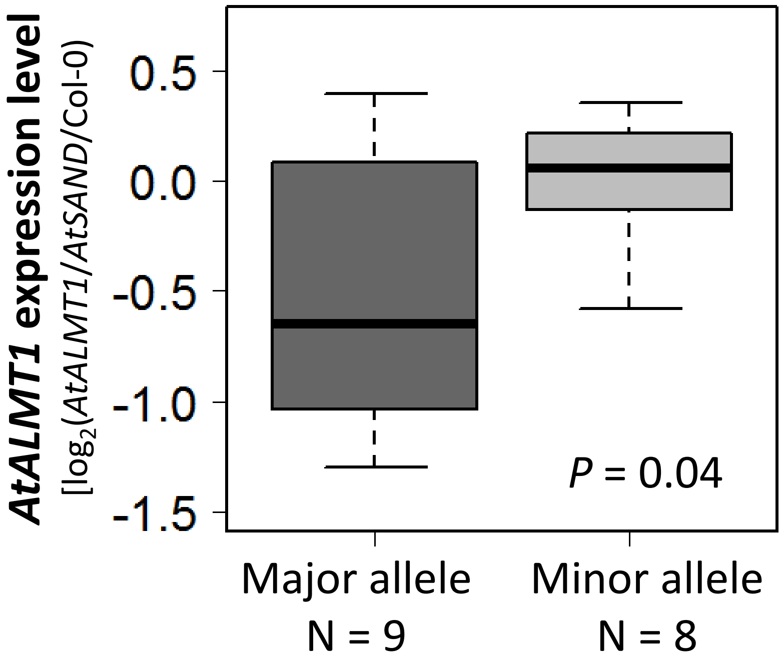

Supplemental Figure S3 A boxplot of *AtALMT1* expression levels of the higher associated single nucleotide polymorphisms (SNPs) on the *AtMATE* locus (Chr.1_19032290, Figure 3). The P-value was calculated by the Student's t-test.

Supplemental Figure S4

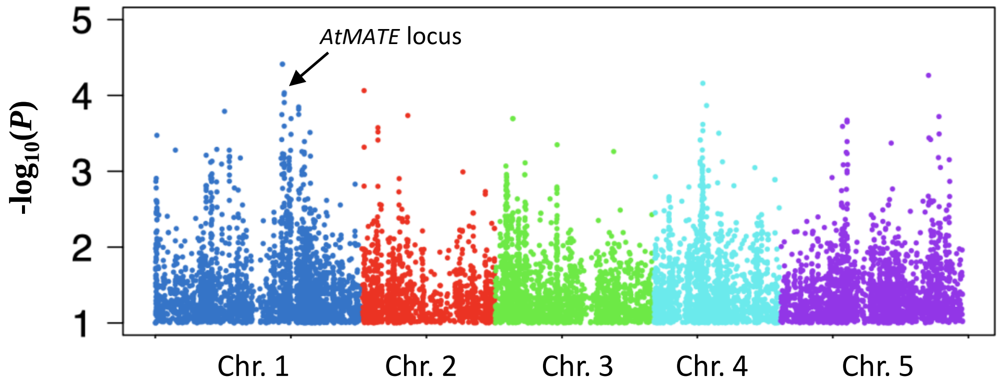

Supplemental Figure S4 Manhattan plot of the genome-wide association study (GWAS) for the *AtMATE* expression level using all testing accession. Only single nucleotide polymorphisms (SNPs) with a p-value < 0.1 are shown.

Supplemental Figure S5

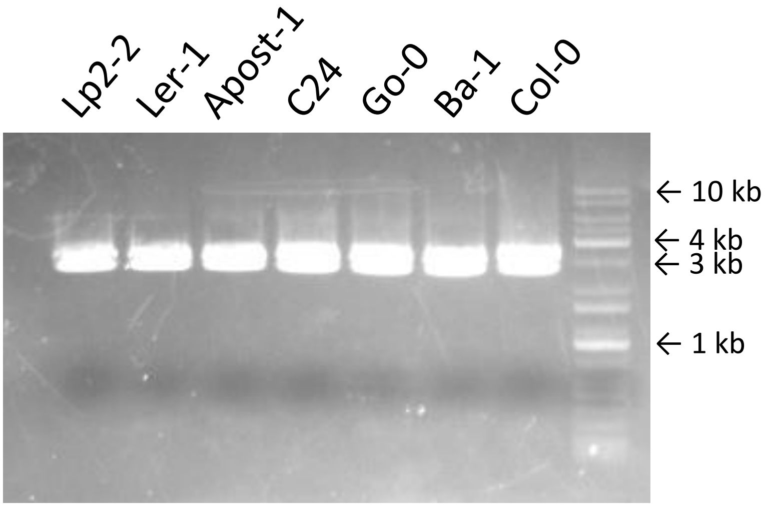

Supplemental Figure S5 Polymerase chain reaction (PCR) amplification analysis of *AtMATE* promoter region for the lower-expression type accessions belonging to other than Central Asia population.

Supplemental Table S1

Supporting Table S1 List of accessions used for this study

Name	*AtMATE* expresssion level	Country	Subpopulation	Latitude	Longitude	Allele of GWAS-detected SNP in *AtMATE* locus		
						Single-population GWAS	GWAS using all accessions	
						Chr1_19032290	Chr1_1902920	Chr1_19033070
Hodja-obi-garm	-1.35	TJK	Central Asia	NA	NA	A	C	C
Kly-4	-2.24	RUS	Central Asia	51.32	82.55	A	C	C
Sij-4	-1.95	UZB	Central Asia	41.45	70.05	A	C	C
Sij-2	-1.93	UZB	Central Asia	41.45	70.05	A	C	C
Koz-2	-1.91	RUS	Central Asia	51.33	82.19	A	C	C
Sij-1	-1.83	UZB	Central Asia	41.45	70.05	A	C	C
Krazo-2	-1.80	RUS	Central Asia	53.09	52	A	C	C
Shahdara	-1.63	TJK	Central Asia	38.35	68.48	A	C	C
Stepn-1	-1.30	RUS	Central Asia	54.06	60.48	A	C	C
Borsk-2	-1.27	RUS	Central Asia	53.04	51.75	A	C	C
Condara	-1.00	TJK	Central Asia	38.48	68.49	A	C	C
Yeg-1	0.27	ARM	Central Asia	39.8692	45.3622	T	C	C
Shigu-2	0.28	RUS	Central Asia	53.33	49.48	T	G	T
Shigu-1	0.55	RUS	Central Asia	53.33	49.48	T	G	T
Rubezhnoe-1	0.66	UKR	Central Asia	49	38.28	T	G	C
Kz-1	0.85	KAZ	Central Asia	49.5	73.1	T	G	T
Leb-3	0.87	RUS	Central Asia	51.65	80.82	T	G	T
Sorbo	1.15	TJK	Central Asia	38.35	68.48	T	G	T
Kidr-1	-0.06	RUS	Central Asia	51.31	57.56	T	C	C
Zdr-1	0.37	CZE	Eastern Europe	49.3853	16.2544	T	C	C
Bor-1	0.79	CZE	Eastern Europe	49.4013	16.2326	T	C	C
Ct-1	0.80	ITA	Eastern Europe	37.3	15	T	C	C
Dra-1	1.13	CZE	Eastern Europe	49.4167	16.2667	T	C	C
Gr-1	1.14	AUT	Eastern Europe	47	15.5	T	C	C
Lp2-2	1.35	CZE	Eastern Europe	49.38	16.81	A	C	C
Ta-0	-0.32	CZE	Eastern Europe	49.5	14.5	T	C	C
Ler-1	0.35	GER	Eastern Europe	47.984	10.8719	A	C	C
Stw-0	0.36	RUS	Eastern Europe	52	36	T	C	C
Eden-1	1.07	SWE	Northern Europe	62.877	18.177	T	G	C
Bil-7	1.26	SWE	Northern Europe	63.324	18.484	T	G	C
Ts-5	-0.12	ESP	Southern Europe	41.7194	2.93056	T	G	C
Angel-1	0.38	ITA	Southern Europe	38.62	16.17	T	C	C
Ciste-2	0.50	ITA	Southern Europe	41.62	12.87	T	C	C
Lago-1	0.98	ITA	Southern Europe	39.18	16.26	T	G	C
C24	1.42	POR	Southern Europe	40.2077	-8.42639	A	G	T
Bak-2	-0.44	GEO	Southern Europe	41.7942	43.4767	T	G	C
Lerik1-3	-0.06	AZE	Southern Europe	38.7406	48.6131	T	G	C
Lecho-1	0.06	BUL	Southern Europe	41.43	23.5	T	C	C
Apost-1	0.57	ITA	Southern Europe	39.01	16.47	A	C	T
Col-0	0.00	USA	Western Europe	38.3	-92.3	A	C	C
Go-0	0.07	GER	Western Europe	51.5338	9.9355	A	C	C
Ba-1	0.22	UK	Western Europe	56.5459	-4.79821	A	C	C
Gu-1	0.50	GER	Western Europe	50.3	8	T	C	C
Est-1	0.63	RUS	Western Europe	58.3	25.3	T	C	C
Kil-0	0.95	UK	Western Europe	56	-4.4	T	C	C
Star-8	1.05	GER	Western Europe	48.43	8.82	T	G	C
Wei-0	-0.71	SUI	Western Europe	47.25	8.26	T	C	C
Nd-1	-0.63	GER	Western Europe	50	10	T	C	C
Bch-3	0.00	GER	Western Europe	49.5166	9.3166	T	G	C
An-2	0.05	BEL	Western Europe	51.2167	4.4	T	G	C

Supplemental Table S2

Supplemental Table S2 List of primers used in this study

Object	Name		Sequence (5'-)
Amplification for *AtMATE* promoter	AtMATE_-2701	F	GTGTATCGTCATTGACTTACCGAAT
	AtMATE_+51	R	GTATAGAGGGTTTCTTGGAAAATCTG
Sequence analysis for *AtMATE* promoter	AtMATE_TE_end	F	GATCTCGCCTTATTTACGTCATAC
	AtMATE_+51	R	GTATAGAGGGTTTCTTGGAAAATCTG
	AtMATE_-1705	R	ATTTCCTGTATGTGGTGGAGAATC
	AtMATE_TE_start	R	GACATTTGTAAAGGGGACGATAAG
	AtMATE_-438	F	GATAGAAAATTTGTGGTTATAACC
AtMATE promoter::GUS	Sfi1C_AtMATEp_Shigu-1_delition	F	GCTTGGCCTGCTTGGCCTAGTTGACAAAAAAAAAAGTGATTTAC
	Sfi1C_AtMATEp_Shigu-1	F	GCTTGGCCTGCTTGGCCAACTTCAAGTCATCAATAATTTGATGTAAG
	Sfi1C_AtMATEp_Sij4	F	GCTTGGCCTGCTTGGCCTGATCGAATCGAAAAGAGAAGAATGTG
	458_nos-ter_sfi1DR	R	AATTCGGCCGCGACGGCCCCCGATCTAGTAACATAGATGAC
	GUS	F	ATGTTACGTCCTGTAGAAACCCC
	Sfi1C_AtMATEp_Sij4_-TE	F	GCTTGGCCTGCTTGGCCGTAAGTAAATTGTAATGTGTGGTCGG
	AtMATE-GUS-joint	R	GGGGTTTCTACAGGACGTAACATTAAGAGATGTTACTGAAGCTTC
	AtMATE_Sij4_-TE_joint	F	GGAGTCATTTTTTATTAAGTGTAGTTGAC
	AtMATE_Sij4_-TE_joint	R	GTCAACTACACTTAATAAAAAATGACTCC
Expression analysis	HPT	F	GTTGACGGCAATTTCGATGATGCAG
		R	CGATTTGTGTACGCCCGACAG
	AtMATE	F	GCATAGGACTTCCGTTTGTGGCA
		R	CGAACACAAACGCTAAGGCA
	AtSAND	F	GGGACCCCACAAGACTCAATA
		R	CATCTTTTACCCTTTGGCACAC
	GUS	F	CGGAAGCAACGCGTAAACTC
		R	GTGTGAGCGTCGCAGAACATT
	AtALMT1	F	CACAGTTTTACATGACGTTGATAATGAT
		R	TCTTCATGTTTTTCATGGTTTGAGTT
ChIP-qPCR	P1	F	TGATTCTCCACCACATACAGGA
		R	ACCTTTCCGATATGCACCTCT
	P2	F	GGTCATGGCCTCAAGAAGTG
		R	TCGCGCTATCTCTAAACCGA
	Mut-like	F	GATTTACAAGGAATCTGTTGGTGGT
		R	CATAACATAGGTTTAGAGCATCTGC
AtSTOP1 promoter::AtSTOP1-GFP	STOP1 promoter region	F	GCTTGGCCTGCTTGGCCGATTCTTTTCGGTTATCCGATATCTACA
	STOP1 coding region	R	GCTCCTCGCCCTTGCTCACCATGAGACTAGTATCTGAAACAGACTCACC
	GFP	F	ATGGTGAGCAAGGGCGAGGAGC
	Nos ter	R	AATTCGGCCGCGACGGCCCCCGATCTAGTAACATAGATGAC

Supplemental Table S3

Supplemental Table S3 List of the transcription factors which potentially binding to the upstream region of the *AtMATE* promoter

AGI	TF	chrom	Binding site (bp)
AT4G36740	ATHB40	chr1	19030009
AT5G65310	ATHB5	chr1	19030012
AT5G60850	OBP4	chr1	19030085
AT1G64620	AT1G64620	chr1	19030144
AT5G02460	AT5G02460	chr1	19030144
AT2G01940	SGR5	chr1	19030230
AT2G02070	IDD5	chr1	19030233
AT2G02080	IDD4	chr1	19030247
AT1G49480	REM19	chr1	19030299
AT1G49480	REM19	chr1	19030300
AT1G34370	STOP1	chr1	19030418
AT5G39760	ATHB23	chr1	19030455
AT5G65410	ATHB25	chr1	19030500
AT2G18350	ATHB24	chr1	19030502
AT1G34370	STOP1	chr1	19030525
AT1G14687	AtHB32	chr1	19030552
AT3G24120	AT324120	chr1	19030567
AT2G20110	AT2G20110	chr1	19030628
AT5G46350	WRKY8	chr1	19030649
AT4G01250	WRKY22	chr1	19030660
AT2G30250	WRKY25	chr1	19030662
AT1G75240	ATHB33	chr1	19030694
AT5G39760	ATHB23	chr1	19030699
AT5G65410	ATHB25	chr1	19030699
AT2G18350	ATHB24	chr1	19030713
AT5G65410	ATHB25	chr1	19030713
AT2G18350	ATHB24	chr1	19030714
AT4G01250	WRKY22	chr1	19030728
AT1G49480	REM19	chr1	19030753
AT1G49480	REM19	chr1	19030754
AT3G18990	VRN1	chr1	19030756
AT4G38170	FRS9	chr1	19030805
